# The domestic pig as a translational model of hyperoxaluria: a pilot study of acute and chronic sodium oxalate infusion

**DOI:** 10.3389/fphys.2025.1692403

**Published:** 2026-01-21

**Authors:** Tomasz Jacek, Dominika Szkopek, Piotr Wychowański, Janine Donaldson, Kamil Zaworski, Mariusz Strutyński, Siarhei Kirko, Olena Prykhodko, Olexandr Fedkiv, Stefan G. Pierzynowski, Kateryna Pierzynowska

**Affiliations:** 1 National Research Institute of Animal Production, Balice, Poland; 2 Large Animal Models Laboratory, The Kielanowski Institute of Animal Physiology and Nutrition, Polish Academy of Sciences, Jabłonna, Poland; 3 Catholic University of the “Sacred Heart”, Department of Head and Neck and Sensory Organs, Rome, Italy; 4 Department of Interventional Dentistry, Collegium Medicum, Nicolaus Copernicus University, Bydgoszcz, Poland; 5 Department of Physiology, Faculty of Health Sciences, School of Biomedical Sciences, University of the Witwatersrand, Johannesburg, South Africa; 6 Department of Animal Physiology, The Kielanowski Institute of Animal Physiology and Nutrition, Polish Academy of Sciences, Jabłonna, Poland; 7 Anara AB, Trelleborg, Sweden; 8 Department of Biology, Lund University, Lund, Sweden; 9 Division of Food and Pharma, Department of Process and Life Science Engineering, Lund University, Lund, Sweden; 10 Department of Medical Biology, Institute of Rural Health, Lublin, Poland

**Keywords:** calcium oxalate, hyperoxalemia, hyperoxaluria, nephrolithiasis, pig model, sodium oxalate

## Abstract

The purpose of this pilot study was to develop and characterize an *in vivo* porcine model of hyperoxaluria using intravenous infusion of sodium oxalate (NaOx). Two experimental regimens were developed to replicate acute and follow up chronic hyperoxaluria. In the acute model, 3 different doses of 1% NaOx were administered over 15 h, resulting in a dose-dependent increase in plasma oxalate concentration (Cmax: 42.4–122.4 µM) and transient hyperoxaluria, with a return to baseline values 6–8 h after stopping the infusion of NaOx solution. In the chronic model, repeated infusions of NaOx for 7–11 days directly after acute tests led to persistent hyperoxalemia (up to 302.4 µM), clinical deterioration and dose-dependent calcium oxalate (CaOx) deposits in renal tissue (1.85%–9.55% of renal surface area), consistent with impaired renal function. The model represents the key clinical features of both rapidly inducible and reversible hyperoxalemia and hyperoxaluria, as well as the progressive nephrocalcinosis. Due to the physiological similarity between pigs and humans, the proposed porcine model could be considered as a quick and valuable tool for studying the pathophysiology of oxalate excess and testing the efficacy of new therapies to counteract its toxicity.

## 1 Introduction

Hyperoxaluria is a pathological disorder characterized by the increased urinary excretion of oxalates. Under physiological conditions, oxalates are formed as the end products of the hepatic metabolism of glyoxylate, amino acids and vitamins (including glycine, hydroxyproline and vitamin C), and their renal excretion and urinary levels remain constant (less than 40 mg/24 h) (Bhasin et al., 2015; Penniston et al., 2017; De Araújo et al., 2020). Primary hyperoxaluria (PH) is a rare congenital condition caused by abnormalities of glyoxylate metabolism in the liver. On the other hand, secondary hyperoxaluria is associated with environmental factors such as a high oxalate diet and diseases (including diabetes mellitus, obesity or Crohn’s disease) that can lead to excessive oxalate absorption in the intestines (Meydan et al., 2003; Carbone et al., 2018; Waikar et al., 2019). Regardless of the etiology, elevated endogenous or exogenous oxalate concentrations in the blood result in increased urinary oxalate excretion, leading to calcium oxalate (CaOx) crystal formation and precipitation, nephrolithiasis, nephrocalcinosis, and consequently chronic kidney disease (CKD) (Sayer et al., 2004).

Preclinical studies of nephrolithiasis and hyperoxaluria require the use of appropriate animal models, which are essential for understanding the pathophysiology of the diseases and testing potential therapies. The incidence of kidney stones disease (KSD) has increased by about 50% in the past 3 decades, resulting in a global prevalence of 13% (Qian et al., 2022; Lang et al., 2022). Kidney stones can be divided into four groups, of which calcium-based stones, including CaOx, are the most common, with hyperoxaluria cited as a major risk factor (Hanstock et al., 2025). To date, several models of hyperoxaluria have been developed, which differ in terms of method of induction, disease course and animal species. Mice and rats are most commonly used for this purpose, mainly due to their low maintenance costs, availability and genetic engineering capabilities. However, rodent models have significant limitations - their kidneys are mono-pyramidal in structure, and they differ from humans in terms of oxalate metabolism, body size and glomerular filtration rate (GFR) dynamics, among other factors. Moreover, rodents are becoming increasingly inbred, causing their genetic diversity to decrease significantly (Shanks et al., 2009). This has been listed as one reason for caution in extrapolating results to people, who demonstrate higher genetic variety (Penniston et al., 2017; Shanks et al., 2009).

For these reasons, there has been a steadily growing interest in large animal models, especially pigs, which have anatomical and physiological similarities to humans. Pigs have multi-pyramidal kidneys, with each medullary pyramid forming multiple separate papillae, allowing for a more reliable representation of human kidney processes (Bagetti Filho et al., 2008). The physiology of the kidneys, including maximum urine concentration, total renal blood flow, GFR, as well as similar body size and excretory parameters, allow diagnostic procedures to be carried out in a manner analogous to clinical conditions (Penniston et al., 2017; Giraud et al., 2011). In addition, pigs better replicate human oxalate balance physiology, including metabolic pathways in the liver (e.g., lactate dehydrogenase (LDH) and alanine-glyoxylate aminotransferase (AGXT)) and Cl^−^/C_2_O_4_^2-^ transport mechanisms (SLC26A6), which promotes the translation of results (location and severity of CaOx deposits) (Penniston et al., 2017; Wang et al., 2021; Wanders et al., 2025). Furthermore, earlier studies of dietary models in pigs show CaOx deposit accumulation in areas typical for humans (Penniston et al., 2017; Mandel et al., 2004). Therefore, we hypothesized that the domestic pig (*Sus scrofa domestica*) represents a suitable research model of acute and chronic hyperoxaluria induced by intravenous infusion of sodium oxalate (NaOx) solution. The use of these models allows not only quantitative analysis of oxalate levels in plasma and urine, but also histopathological evaluation of the kidneys. Unlike substances that require metabolism or take a long time to have an effect, including ethylene glycol (EG) and L-hydroxyproline (HYP), NaOx is a direct source of oxalate, and its intravenous administration results in an immediate increase in its plasma concentration. By assessing both the course of rapidly inducible hyperoxalemia and hyperoxaluria, and long-term changes in the organ’s structure, these models provide a better understanding of oxalate-dependent mechanisms of kidney injury and can be used to evaluate new therapeutic strategies.

## 2 Materials and methods

### 2.1 Animals, housing conditions, surgical procedures

#### 2.1.1 Bioethics

All experimental procedures were approved by the Malmö/Lund Ethics Review Committee on Animal Experiments, Lunds city court (Malmö/Lunds djurförsöksetiska nämnd, Lunds tingsrätt, Box 75, 221 00 Lund, Sweden, approval number M73-15). The research and care of animals was carried out in accordance with the principles for the care and use of experimental animals (Directive 2010/63/EU of the European Parliament and of the Council of 22 September 2010 on the protection of animals used for scientific purposes). The clinical condition of the animals, including humane end points (loss of appetite, apathy, lack of activity, vomiting, oliguria/anuria), was assessed daily throughout the experiment.

#### 2.1.2 Animals and housing

Two experiments were conducted on a total of 9 castrated male hybrid pigs ((Swedish breed × Yorkshire breed) × Hampshire breed), with an average age of 12 ± 2 weeks and an initial average weight of 12.1 ± 0.8 kg obtained from the Odarslöv research farm. Animals were maintained on a 12-h day-night cycle, temperature 21 °C–25 °C, air exchanges 10–12/h and humidity 70% ± 5%. The pigs were individually housed in 1.0 × 2.0 pens equipped with a dry feeding trough, a drinking nipple and a constant heating lamp (150 W, 24 h). They were allowed to move freely within their pens and had visual contact with each other.

#### 2.1.3 Feed

All pigs were fed a grain-based feed for young growing pigs (53908 VÄXTILL 320 P BK, Lantmännen, Sweden). This diet is rich in calcium and magnesium, containing 61.4% carbohydrates, 17.6% crude protein, 12.4% water, 3.9% crude fiber, 3.5% crude fat and 5.1% ash, along with 0.46% calcium carbonate and 0.82 monocalcium phosphate. The daily feed intake was recalculated once a week, after the pigs were weighed, and was 4% of the animal’s current body weight, which corresponds to the amount eaten under standard pig feeding conditions. Pigs were fed twice a day at 09:00–10:00 and 17:00–18:00 (2% of bwt/meal).

#### 2.1.4 Implantation of central access to the external jugular vein

Before surgery, the animals were premedicated intramuscularly with azaperone (2 mg/kg bwt) (“Stresnil,” Janssen Pharmaceutica, Beerse, Belgium) and ketamine (20 mg/kg bwt) (“Ketalar,” Parke-Davis, Morris Plains, New Jersey). The animals were anesthetized with a mixture of 1.5%–3.0% isoflurane (Forene®, Abbot AB, Sweden), using a semi-open Komensaroff inhalation anesthesia system with an oxygen flow of 0.5 L/min (Medical Developments, Australia). The incision line was epidurally anesthetized with a 2% lidocaine solution (1 mg/kg bwt) (“Xylocaine 2%,” Aspen Pharma, Durban, RPA).

The procedure was performed under aseptic conditions. Cannulas made of a 15–20 cm long silicone tube with a wall thickness of 0.22 mm (outer diameter 1.69 mm; inner diameter 1.47 mm) were inserted into the animals’ external jugular vein. Halfway through their length, the cannulas are equipped with 4 stops made of silicone glue, spaced approximately 1 cm apart. The incision was made along the angle of the mandible - shoulder joint. After dissecting the maxillary vein, just before its transition into the external jugular vein, a small (approximately 2 mm) incision was made. Then, a cannula was inserted into the vessel lumen to a depth of approximately 10 cm towards the heart, up to the first silicone stop. The vessel above the cannula was closed. The remaining stops were used to stabilize the long arm of the catheter in the tissues, which were inserted under the skin in the neck area approximately 7 cm from the base of the ear. Before suturing the surgical wound, ampicillin (Doctacillin®, Astra, Sweden) was administered topically at a dose of 25 mg per animal and buprenorphine (Tamgesic®, Schering-Plough, Belgium) at a dose of 15 mcg/kg bwt intramuscularly. The surgical wound was sutured with a mattress suture using size 0 non-absorbable sutures (Silk, Ethicon, Johnson and Johnson, Great Britain), which were removed 10 days after the procedure.

In the postoperative period, daily and systematic assessment of clinical condition was performed (food and water intake, motor activity, response to stimuli, posture and locomotion, respiratory rate (RR), respiratory effort, heart rate (HR), mucosal color, capillary refill time (CRT), body temperature, diarrhea, vomiting, postoperative wound condition) on a point scale. In addition, the animals were assessed daily on modified Unesp-Botucatu pain scale by two independent veterinarians. The patency of the external jugular vein access was also assessed daily (flushing with heparinized 0.9% NaCl, changing the wound dressing).

### 2.2 Experimental design and sample collection

#### 2.2.1 Acute experiment

**FIGURE 1:**
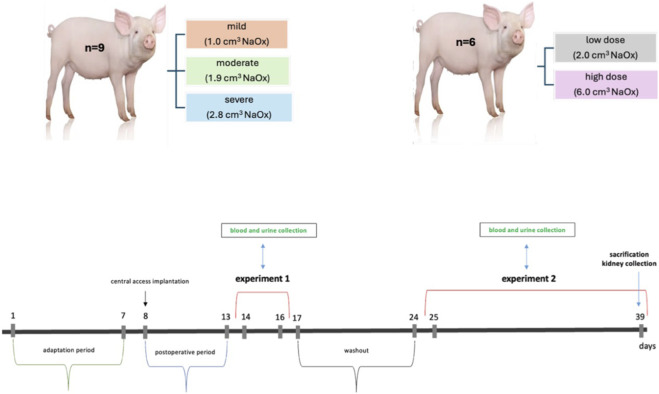
Research groups in acute (experiment 1) and chronic (experiment 2) and a simple study design.

The experiment was conducted on 9 pigs, which were divided into 3 experimental groups (n = 3 per group): mild, moderate and severe hyperoxalemia (Figure 1). Sodium oxalate solution (Sigma-Aldrich Chemicals, St. Louis, MO, United States) was prepared using sterile 0.9% NaCl to obtain a 1% isoosmotic solution. The solution was sterile filtered and administered intravenously by bolus injection of 1 mL (mild), 1.9 mL (moderate) or 2.8 mL (severe) via the jugular vein, every 15 min during a 15-h period to obtain plasma oxalate concentrations similar to those observed in patients with hyperoxalemia and oxalosis (>40 mg/24 h).

Five mL blood samples were collected from each animal 1 hour before the start of the experiment, 1 h after the start of the infusion and every 4 h during the infusion. Blood samples were also collected after infusion (at timepoint 20 and 24 h). Collected samples were transferred immediately into tubes with clotting activator (BD Vacutainer SST™, United States) and centrifuged (Multifuge 1, Heraeus, United Kingdom) at 3,000 × g for 15 min at 4 °C. The collected serum was stored in 1 mL aliquots at −20 °C to determine the oxalate concentration per liter of blood using ion chromatography.

The animals’ daily urine was collected for 24 h before the start of the experiment, during the infusion and then at 9 and 24 h after the end of the infusion. Urine was acidified with 6N HCl to avoid the proliferation of bacterial flora and the formation of oxalate deposits. Urine volume was measured and then frozen (−22 °C ± 2 °C) to determine oxalate concentration. After 7 days from the end of experiment 1, 6 pigs were randomly chosen to be used in experiment 2.

#### 2.2.2 Chronic experiment

The experiment was conducted on 6 pigs, which were divided into 2 experimental groups (n = 3 per group) (Figure 1). Sodium oxalate (Sigma-Aldrich Chemicals, St. Louis, MO, United States) was made as a sterile 1% sodium oxalate concentration in 0.9% NaCl. The 1% sodium oxalate solution was infused into the external jugular vein by cyclically injecting a specified amount of the solution: 6 mL every 30 min in 3 pigs (high dose) and 2 mL/30 min in the other 3 pigs (low dose). The infusion time of the oxalate solution was 24 h and lasted for a maximum of 11 days.

To monitor the level of hyperoxalemia, blood samples were collected several times (0, 7, 11 and 14 days) during the 2 weeks of experimental period. Similar to experiment 1, the collected blood samples were prepared and stored at −20 °C until further analysis by ion chromatography. During the experiment, the animals’ feed intake and clinical condition were monitored daily.

#### 2.2.3 Autopsy

At the end of the experiment, animals were sacrificed with single dose of sodium pentobarbital intravenous injection (140 mg/kg bwt) (Allfatal vet. Omnidea AB, Sweden) and kidneys were collected for histological analysis.

### 2.3 Ion chromatography

An ICS- 900, CO-DV ion chromatograph (Thermo Scientific, MA, United States) was used to analyze oxalate concentrations in all samples. Serum was filtered through 3 kDa centrifugal filters (VWR International, Stockholm, Sweden). Urine samples were acidified with 6N HCl to reach pH values <2. Urine samples were also filtered through 3 kDa centrifugal filters (VWR International, Stockholm, Sweden). For ion chromatography the following columns were used: IonPack AG4A-SC (2 × 50 mm), IonPack AS4A-SC (2 × 250 mm), AMMS 300 supressor, mobile phase 1.8 mM Na2CO3/1.7 mM NaHCO3, flow rate 0.5 cm^3^/min, regenerant 75 mN H2SO4.

### 2.4 Histological evaluation of kidneys

10 3-cm kidney fragments were collected from each animal and fixed in 10% neutral buffered formalin for 24 h. Then, the tissue fragments were dehydrated in ethanol of increasing concentrations (50%, 70%, 80%, 90%, 96%, 99.8%) and embedded in paraffin according to standard histological techniques. Paraffin-embedded tissues were cut into 4–5 μm thickness sections using a rotor microtome and were evaluated using the Yasue method (Evan et al., 2003; Canela et al., 2021). The analysis was performed using a light microscope (Axioskop 40, Zeiss, Jena, Germany) equipped with a digital camera (Coolpix B700, Nikon, Tokyo, Japan). All tissue samples were analyzed by a public domain Java image-processing program, ImageJ v.1.46 (National Institute of Health (NIH), Bethesda, Maryland, United States). The result is expressed as % of CaOx deposits in the tissue and presented as the sum of % CaOx positive areas from the cortex and medulla for each kidney. The severity of the nephrocalcinosis was graded using arbitrary units. Nephrocalcinosis was considered low if ≤1% of the kidney area was affected, moderate if 1%–3% of the area was affected and severe if ≥ 3% of the area was affected.

### 2.5 Statistical analysis

GraphPad Prism 10.5.0 (San Diego, CA, United States) was used for statistical analyses. Data were tested for normal (Gaussian) distribution using the Shapiro–Wilk normality test. Differences in investigated parameters were assessed using an unpaired t-test (data on plasma oxalate in High Dose Group, Chronic Experiment) or one-way ANOVA for the normally distributed datasets. For the data with non-Gaussian distribution (oxalate deposition in kidney tissue) the Mann-Whitney test was used. Normally distributed data are shown as Mean ± SD (standard deviation), while data with non-Gaussian distribution are represented as Median ± IQR (interquartile range). Differences were considered significant if p < 0.05.

## 3 Results

### 3.1 Serum oxalate concentration in acute study

**FIGURE 2:**
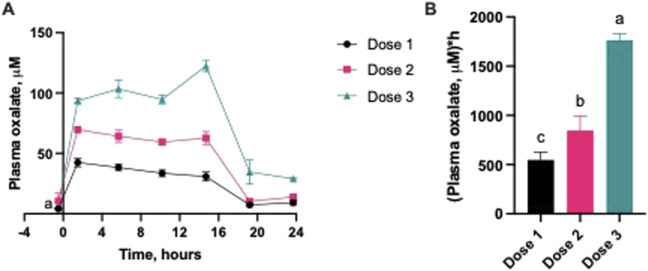
Blood oxalate profile from pigs with mild to severe hyperoxalemia. **(A)** Changes over infusion time and after oxalate removal; **(B)** Area under the curve comparison. Dose 1 - 1 mL, Dose 2 - 1.9 mL, Dose 3 - 2.8 mL of 1% sodium oxalate via jugular vein every 15 min during a 15-h period (n = 3 per group). Results are presented as mean ± SD. Area under the curve (AUCs) values are baseline-adjusted. Significant differences are indicated by different letters (a, b, c; *p* < 0.05).

The level of oxalate in the blood increased steadily and was dependent on the concentration of oxalate infused to the pigs (Figure 2A). A significant increase in plasma oxalate levels was observed following oxalate infusions, regardless of dose, with maximal concentration (Cmax) reaching 42.4 ± 3.5, 69.6 ± 0.2 and 122.4 ± 4.9 μM following infusion with the low, medium and high dose, respectively. Plasma oxalate levels were generally stable 1 h after the start of the infusion, until the end (14.5 h). A clear significant dose-dependency was observed in the area under the curves (AUC) of the plasma oxalate levels reached and the dose of oxalate infused (Figure 2B). Plasma oxalate levels returned to baseline values ca 6–8 h after the oxalate infusion was stopped.

### 3.2 Urinary oxalate concentration in acute study

**FIGURE 3:**
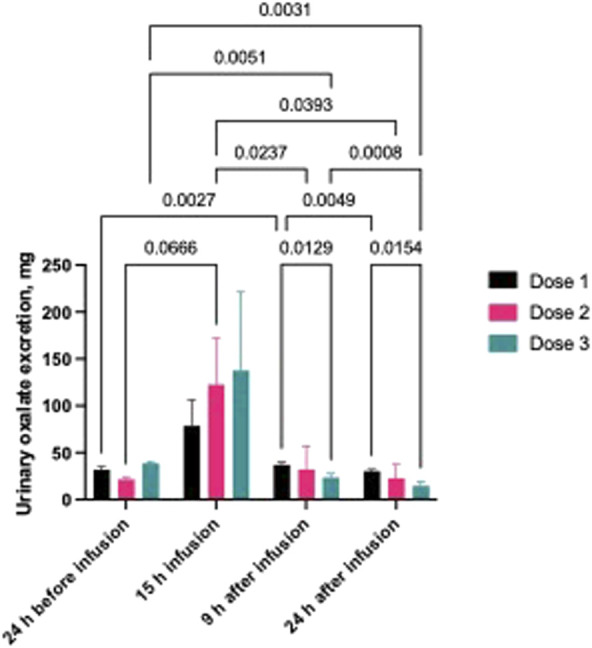
Urinary oxalate concentration in pigs with mild to severe hyperoxalemia. Dose 1 - 1 mL, Dose 2 - 1.9 mL, Dose 3 - 2.8 mL of 1% sodium oxalate via jugular vein every 15 min during a 15-h period (n = 3 per group). Results are presented as mean ± SD.

Urinary oxalate excretion was also monitored before, during and after oxalate infusion. As shown in Figure 3, oxalate excretion peaked during the 15-h continuous oxalate infusion and was not dependent on the concentration of oxalate infused to the pigs. Urinary oxalate returned to baseline levels within 24 h following the end of the oxalate infusion.

### 3.3 Serum oxalate concentration during chronic NaOx infusion

**FIGURE 4:**
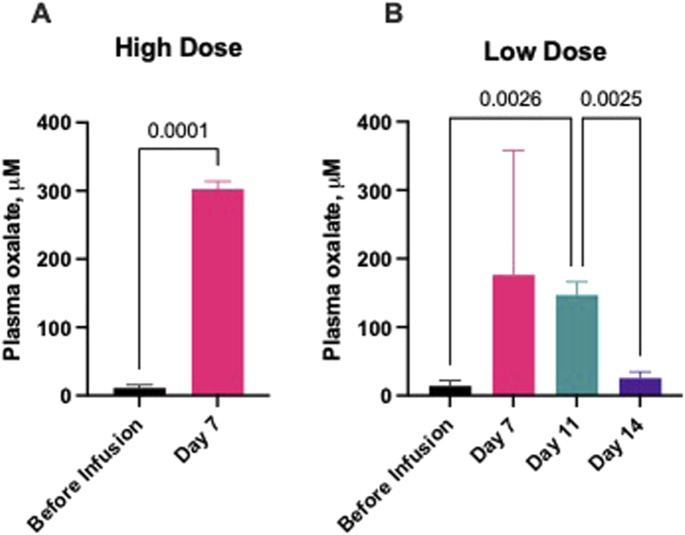
Serum oxalate concentration during sodium oxalate infusion. **(A)** High Dose (n = 3) and **(B)** Low Dose group (n = 3). Blood samples were taken 24 h before the start of the oxalate infusion and on 7th, 11th and 14th day of the study. Pigs were infused with 1% sodium oxalate at 2 mL every 30 min in the Low Dose Group, and at 6 mL every 30 min in the High Dose group, respectively. Oxalate infusions were stopped in the High Dose group after 7 days and in the Low Dose Group after 11 days. Plasma oxalate levels were additionally measured 3 days after oxalate infusion was stopped (day 14 results). Results are presented as mean ± SD.

Pigs receiving the “high dose” oxalate infusion were infused with 6 mL of 1% NaOx solution every 30 min. Serum oxalate concentration increased rapidly, reaching 302.4 ± 11.46 μM on the 7th day (Figure 4). The deteriorating health condition of the pigs, including lack of appetite, reduced water consumption, significantly reduced activity, apathy, vomiting, and no or minimal diuresis, did not allow the study to continue. The pigs were euthanized on the 7th day.

Pigs receiving the “low dose” oxalate infusion were infused with 2 mL of 1% NaOx solution every 30 min. After 7 days, the serum oxalate concentration reached 176.1 ± 181.7 uM (Figure 4). The continuous low dose oxalate infusion also induced severe hyperoxalemia and serum oxalate levels on day 11 were in the range of between 130.9 and 167.9 uM (Figure 4). The oxalate infusions were stopped on day 11 in pigs from this group as a result of the poor health condition of the pigs, which manifested as weakness and vomiting.

### 3.4 Daily feed intake in a chronic study

**FIGURE 5:**
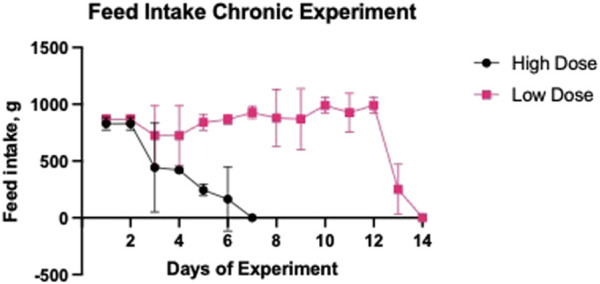
Daily feed intake during low and high dose oxalate infusions. Pigs in the Low Dose group were infused with 1% sodium oxalate at 2 mL every 30 min and pigs in the High Dose group were infused with 1% sodium oxalate at 6 mL every 30 min. Oxalate infusions were stopped in the High Dose group after 7 days. Results are presented as mean ± SD.

The amount of feed consumed by the pigs was measured throughout the experimental period (Figure 5). In the pigs receiving the Low Dose infusion, there was a significant reduction in feed intake by 74.6% on day 13 and by 100% on day 14, compared to the recommended feed intake (990 g). In pigs receiving the High Dose, oxalate infusion conducted at a rate of 6 mL/30 min resulted in a significant decrease in feed intake, with a 49.3% reduction on day 3, 70.7% on day 4, 80.4% on day 5, and 100% on day 6 of the experimental period.

### 3.5 Oxalate deposits in the kidney tissues

**FIGURE 6:**
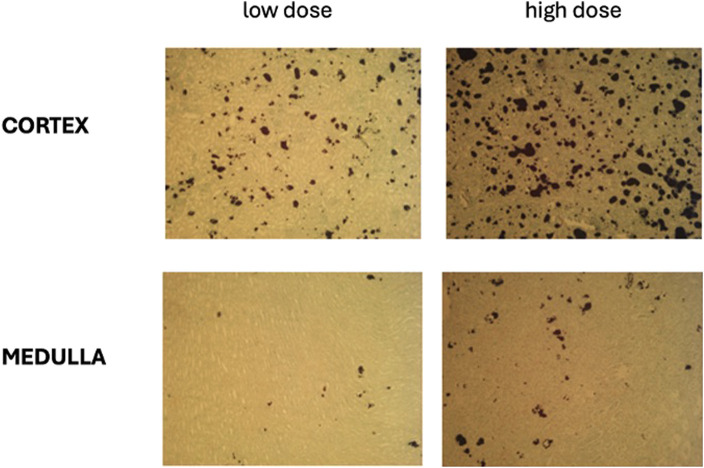
Representative images of nephrocalcinosis in the renal cortex and medulla of pigs from the Low and High dose NaOX groups. The black staining represents CaOx deposits. Yasue staining, low magnification (X25).

**FIGURE 7:**
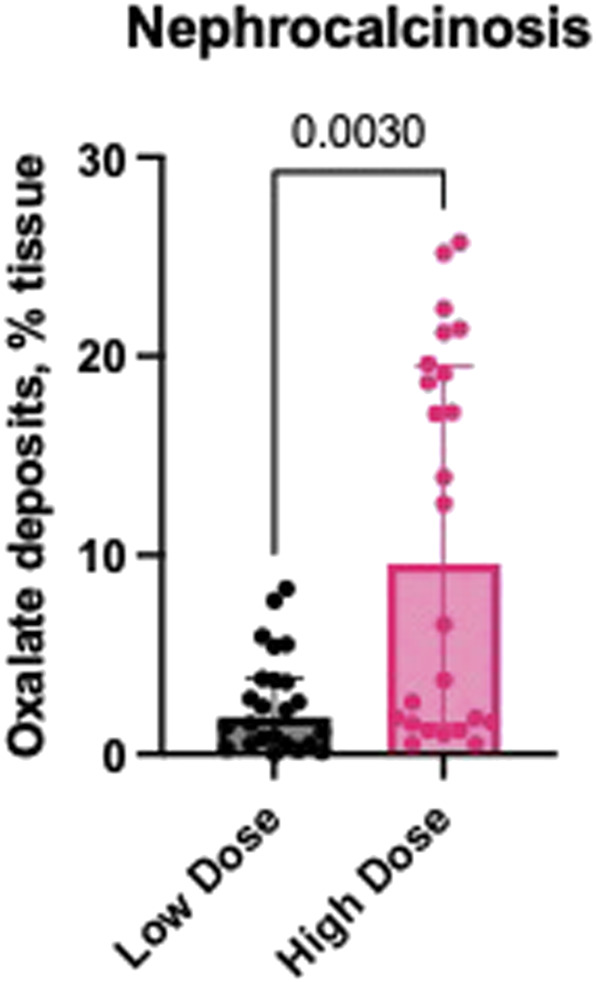
Percentage nephrocalcinosis in kidney tissues of pigs from the Low and High Dose NaOX groups (Median (±IQR). Yasue staining for CaOx deposits was performed in representative samples from the right and left kidneys and affected areas were calculated as 2 independent areas from the cortex and 2 independent areas from the medulla, with a total of four images per kidney (L or R). Low magnification (X25).

Each histologically evaluated sample contained renal cortex and medulla from 2 different parts of each kidney (left and right, Figure 6). Figure 7 shows the median percentage of calcium oxalate in the kidneys of pigs from the High Dose group after 7 days of oxalate infusion, which was 4 times higher than that observed in pigs from the Low Dose group after 11 days of infusion and 3 washout days. The median oxalate deposition in the kidney tissues of pigs from the High Dose Group reached 9.55%, which could be considered as severe nephrocalcinosis, while in the Low Dose Group the median percentage of oxalates was 1.85%, which could be considered as moderate nephrocalcinosis (Figure 7).

## 4 Discussion

The aim of the acute experiment was to evaluate the effectiveness of a porcine model in reproducing acute hyperoxalemia and hyperoxaluria using an intravenous infusion of 1% NaOx. The intravenous administration of NaOx effectively induced acute hyperoxalemia in the pigs in a dose-dependent manner, reflected both in the Cmax and AUCs values of plasma oxalate, which were stable and reversive. Previous studies on hyperoxalemia mainly make use of animal models which require prolonged exposure to the agent and do not reflect the sharp and rapid increases in plasma oxalate concentrations (POx) observed in the present study. Ethylene glycol (EG), often used in rodent models, is metabolized in the liver, indirectly yielding oxalate, but its toxicity to other organs (especially the liver and CNS), as well as the risk of metabolic acidosis, limits its use (Tzou et al., 2016). Verhulst et al. (2022) used EG in drinking water (0.75%) to induce primary hyperoxaluria (PH) in a rat model, leading to a significant increase in POx. In contrast to our study, the level did not significantly increase until week 2 of the experiment, and a plateau was not reached until week 6. Oxalate exposure can be achieved with a high oxalate diet, as in the study by Crestani et al. (2021), but this method requires a longer time to induce hyperoxalemia and there is a high variability in the bioavailability of dietary oxalate. The method of intravenous infusion of NaOx used in our study, as a direct source of oxalate, allows precise control of oxalate concentration in the blood and its duration of action, and the rate of reaching a plateau within 1 h after the start of infusion indicates high bioavailability and immediate action. Our results are in agreement with an earlier study by De Araújo et al. (2020) in a rat model in which administration of NaOx (7 mg/100 g i.p.) was shown to cause features of acute kidney injury (AKI), including inflammatory infiltration and degeneration of renal tubular epithelium, within 24 h.

Another important aspect of the presented study is the parallel analysis of urinary oxalate excretion, which confirms the physiological renal response to hyperoxalemia. Despite the differences in dosing in the acute model, peak oxalate excretion was similar between the groups and occurred mainly during the oxalate infusion but returned to baseline levels within 24 h after the infusion ended. This is consistent with a study by Penniston et al. (2017) in a porcine model in which hyperoxaluria was induced by feeding the pigs a diet containing hydroxyproline (HYP). The authors observed a dynamic response in pigs to the administration of exogenous oxalate in the form of increased urinary oxalate excretion and a rapid return to homeostasis/baseline levels. In a study by Kaplon et al. (2010), the addition of HYP to the diet of pregnant sows also led to an increase in urinary oxalate excretion, with a peak on day 3 and a gradual return to baseline on day 6. Khan et al. (1979), Khan et al. (1981), Khan et al. (1991) reported an increase in urinary oxalate excretion after a single injection of NaOx in mice and rats, followed by a decrease over the next 12 h. In humans, a similar phenomenon occurs under physiological conditions, where the kidneys efficiently regulate plasma oxalate levels through glomerular filtration and tubular secretion, the latter mediated by the SLC26A anion exchanger and the Cl^−^/oxalate exchanger SLC26A6, unless they are damaged (Robijn et al., 2011; Kn et al., 2019; Fargue et al., 2023). This result indicates a preserved compensatory capacity of the excretory system under controlled and brief exposure to NaOx.

The purpose of chronic experiment was to evaluate the effectiveness of the porcine model in reproducing chronic hyperoxaluria using repeated intravenous infusions of 1% NaOx. In the High Dose group, maximum POx reached was 302.4 ± 11.46 µM after just 7 days of infusions, while in the Low Dose group the values were slightly lower, but still well above physiological norms. It is accepted that a decrease in GFR below 30–40 mL/min per 1.73 m^2^ of body surface area results in impaired renal excretion of oxalate and an increase in plasma oxalate concentration (normal limit 1–6 μM/L) (Hoppe et al., 1999) and can rapidly exceed the supersaturation threshold for calcium oxalate as levels >30 μmol/L are reached (Hoppe et al., 2009). According to Perinpam et al. (2018), in the diagnosis and monitoring of PH, enteric hyperoxaluria and urinary stone disease, in addition to parameters such as GFR or urinary oxalate excretion, POx should also be considered. In PH patients in end-stage renal disease (ESRD), plasma oxalate levels are usually higher than 80 μM/L, while in non-PH hyperoxaluric patients, the POx level may range between 30 and 80 μM/L (Bhasin et al., 2015). The increase in POx observed in our study is also consistent with clinical studies in humans (Hoppe et al., 1999), whereas significantly higher mean POx was observed in children with PH1 than in those without PH. Nevertheless, the POx values presented in our chronic study not only exceed the levels observed in acute clinical conditions but are also consistent with the range noted in PH1 patients and exceed toxicity thresholds posing a high risk of developing systemic oxalosis, especially in ESRD patients (Bhasin et al., 2015; Hoppe et al., 1999). The relationship between chronically elevated oxalate levels and the progression of CKD has been confirmed in population-based studies, where it was found that higher urinary oxalate excretion (upper quintile ≥27.8 mg/24 h) significantly correlated with a more rapid decline in renal function and higher risk of ESRD (Waikar et al., 2019). Moreover, this was confirmed in the present study by clear clinical signs, especially in pigs in the High Dose groups, including apathy, vomiting and decreased appetite, with complete cessation of food intake. In studies on a rat model with induced hyperoxaluria, Verhulst et al. (2022) and Crestani et al. (2021) also found a significant reduction in food intake, but without such clear clinical signs as in our study. These symptoms correspond to those of the cachexia syndrome observed in the late stages of CKD and ESRD in humans. Thus, it can be concluded that this model allows modulation of the severity of changes depending on the dose and duration of infusion, which has important implications for studies on the toxic effects of oxaliplatin, as well as on the efficacy of interventional therapies.

This study shows that intravenous administration of NaOx leads to markedly different CaOx deposition in the kidneys, depending on the dose used. In the High Dose group, CaOx deposits occupied an average of 9.55% of the renal cross-sectional area, which can be classified as severe nephrocalcinosis. In the Low Dose group, the percentage was 1.85%, corresponding to moderate lesions. The significantly higher retention of CaOx crystals in the High Dose group is consistent with the higher POx levels in this group. A similar relationship between deposit accumulation and the severity of renal parenchymal damage has been observed in humans. Sayer et al. (2004) showed that the presence of CaOx deposits in renal tubules correlates with epithelial damage, inflammatory infiltration and interstitial fibrosis, which can lead to permanent deterioration of renal function. This is consistent with our results, as the High Dose group not only had a higher percentage of the kidney surface occupied, but also clear clinical signs indicating deteriorating organ function. Additionally, under conditions of high POx, CaOx crystallization occurs within the renal tubules, particularly in their distal segments and in the renal medulla. A study by Khan (2010) confirms that conditions in the terminal sections of the nephron (low pH, high calcium concentration, slower urine flow) promote the formation of deposits, which consequently leads to an inflammatory response and the initiation of fibrosis. Fargue et al. (2023) showed that the presence of CaOx correlates with increased expression of pro-inflammatory cytokines and fibrosis mediators, leading to progressive loss of organ function. In our study, CaOx was found in both the cortex and medulla of the kidney, confirming that its distribution resembles that observed in humans (Penniston et al., 2017). A study by Mehr et al. (2021) in which rats were given NaOx (2.5%) along with water for 4 weeks also found CaOx deposition in the kidneys, but the percentage of kidney surface area occupied by deposits was much lower than in our study. In contrast, in a study by Mandel et al. (2004), in which pigs were fed HYP for 20 days, the formation of calcium oxalatepapillary deposits were observed, which may be precursors of kidney stones. In contrast to both studies, in our study we found kidney stones as early as day 7 (High Dose group) and day 14 (Low Dose group), greatly reducing the duration of the study and confirming the usefulness of this model for assessing the effects of oxalate toxicity on a macro- and microscopic scale.

Finally, we would like to point out that this was a pilot study which definitely had some limitations. Firstly, a small number of animals used in each group, which was done purposely to follow the 3R principle (Replacement, Reduction, Refinement) considering the pilot character of the study. Secondly, the lack of a control group, e.g., with saline infusion, which could be explained by the fact of using baseline values (before infusion) from each animal as a reference point, which allowed both for group- and intra-individual comparisons. Moreover, the existing pool of knowledge on hyperoxaluria and hyperoxalemia development does not allow considering the saline injections as the potential source of oxalate. Finally, the lack of additional analyses of kidney function, including creatinine, urea, and electrolyte concentrations (e.g., blood calcium level analysis, calcium-phosphorus metabolism, urine ionic composition), specification for crystal form (monohydrate vs. dihydrate CaOx), as well as advanced histopathological evaluation, including assessment of interstitial damage, fibrosis (IF/TA), and glomerular changes, could be also considered as the limitation of the presented study. However, one should remember that the main objectives of the study were the development of hyperoxalemia and hyperoxaluria, as well as the nephrocalcinosis initiation. All the mentioned endpoints were reached. As no specific drug for reducing/treatment of hyperoxaluria was tested, the additional pool of analysis was considered unnecessary. It should also be mentioned that the high dose of NaOx used in the chronic study may not be suitable for long-term studies, since the comparison of described method with other methods such as HYP feeding or a high-oxalate diet, has never been done.

The results we presented here confirm that the porcine model allows us to reproduce both acute and chronic changes associated with hyperoxalemia and hyperoxaluria. The use of 1% NaOx not only allows us to induce nephrocalcinosis in a reproducible and dose-dependent manner, but also to study its functional and pathophysiological consequences. The NaOx intravenous infusion model effectively mimics hyperoxalemia—a common endpoint in both primary and secondary hyperoxaluria—and allows POx concentrations to be maintained within clinically relevant ranges. In addition, the pig, due to its similar anatomy and physiology to humans, provides a valuable model for translational research into new therapies for hyperoxalemia and hyperoxaluria and toxicity associated with excess oxalate.

## Data Availability

The original contributions presented in the study are included in the article/supplementary material, further inquiries can be directed to the corresponding authors.
